# Exploring Personality Traits, Values, and Attitudes toward Professionalism: Implications for the Promotion of Mental Health and Functioning in Medical Students

**DOI:** 10.3390/healthcare12171732

**Published:** 2024-08-30

**Authors:** Polona Selič-Zupančič, Davorina Petek, Nina Jerala

**Affiliations:** 1 Department of Family Medicine, Faculty of Medicine, University of Ljubljana, Poljanski Nasip 58, 1000 Ljubljana, Slovenia; polona.selic@mf.uni-lj.si (P.S.-Z.); davorina.petek@mf.uni-lj.si (D.P.); 2 Department of Psychology, Faculty of Medicine, University of Maribor, Taborska 8, 2000 Maribor, Slovenia; 3 University Psychiatric Clinic Ljubljana, Studenec 48, 1260 Ljubljana, Slovenia

**Keywords:** medical students, mental health, Professionalism Attitude Scale, professionalism, personality traits, personal values, medical education, Big Five Questionnaire

## Abstract

Healthcare workers face significant mental health challenges, including stress, burnout, and psychological distress, leading to high rates of mental health symptoms and even suicide attempts, as well as an increase in medication errors and unprofessional behavior. Targeted interventions are needed to address these issues. However, promoting healthier traits in medical students or refining selection could also prove beneficial, as research shows that mental health is significantly influenced by personality traits and personal values. This study examines the relationship between personality traits, values, and attitudes toward professionalism among medical students in Slovenia. A total of 996 participants were examined in three data collections from the academic years 2015–2016 to 2019–2020 using the Big Five Questionnaire, the Personal Values Scale, and the Attitude Toward Professionalism Scale. Hierarchical linear regression analysis was conducted to examine the factors associated with professionalism. The results showed that attitudes toward professionalism were stable over the years, with higher scores consistently associated with the female gender, agreeableness, and conscientiousness. Conversely, material value orientation had a negative impact on professionalism. In addition, we examine the associations between mental health and personality traits, personal values, and attitudes toward professionalism to illustrate the importance of selecting and nurturing medical students, based on traits that promote mental health and professional behavior. These findings may lead to improvements in medical education and selection processes to improve the well-being and functioning of future medical professionals.

## 1. Introduction

A growing body of data suggests that healthcare workers face significant mental health problems, such as high levels of stress, burnout, and psychological distress, and as a result have high rates of mental health symptoms, suicidal thoughts, and even suicide attempts [1]. The overall prevalence of burnout [2], depression, suicidal thoughts [3], and stress [4] is already higher in medical students than in the general population. The demanding nature of the healthcare sector, which is characterized by long working hours, a high volume of patients, and a high administrative burden, also contributes to considerable stress and later burnout among doctors. Some studies show that up to 50% of healthcare workers have experienced stress or burnout in recent years [5]. The impact of COVID-19 has further exacerbated the mental health challenges faced by healthcare workers [6]. A study published in 2023 found that healthcare workers experienced increased burnout and mental health problems one year after the outbreak of the COVID-19 pandemic. This study showed that around 52% of healthcare workers reported burnout after three months, which remained relatively stable at 51% after twelve months. This persistent burnout highlights the ongoing stress and mental health challenges in this profession [7].

Burnout is the best-known inverse metric of physician well-being and has been shown to have personal consequences; hospitals characterized as having too few nurses and unfavorable work environments had higher rates of clinician burnout and turnover, as well as unfavorable patient safety ratings [8]. Therefore, professionalism becomes one of the most important competencies that all medical students should acquire during their training [9], especially when they are exposed to an increasingly clinical curriculum and routine patient interactions during the course of their medical studies. Ironically, it is at this time that students’ attitudes toward pro-professionalism tend to decline, as older students have a more negative view of primary care, physician status and income, and lower levels of idealism [10,11]. In addition to year of study, the studies also show that mental health has an important relationship with professional attitudes. Students with good mental health were more likely to have a greater number of the five altruistic professional beliefs related to physicians’ responsibility to society [12,13].

Personal resources act as a buffer against stress, burnout, and negative emotions in connection with the challenges of the medical profession. For example, one study examined how negative emotions (such as fear and anxiety) affect nursing students’ commitment to the nursing profession (commitment is an important aspect that affects professionalism) and how students with higher levels of psychological capital (which includes elements such as resilience, optimism, and self-efficacy) were better able to maintain their commitment despite the challenges posed by the pandemic [14]. Similarly, innate personality traits, which are 43% to 54% heritable [15], provide greater psychological resilience [16]. A comprehensive review of studies, including a metasynthesis of published research, also clearly shows that all Big Five personality traits are strongly associated with general health, particularly mental health [17], and subjective and psychological well-being [18]. This influence can be transferred to mental health, both directly and indirectly, through various domains such as career success, social support, relationship stability, resilience, mitigation of burnout, and adaptive coping strategies [19,20,21,22,23,24,25,26,27,28]. Some personality traits, of which conscientiousness is the most important, are strongly associated with professionalism [29] and academic success [23], both important factors for medical students. Previous studies have examined the personality dimensions of medical students. They scored relatively high on extraversion and conscientiousness, but were mid-range on conscientiousness, neuroticism, and openness [30].

Personal values, which we consider to be the result of early learning with influences from society and family of origin, are known to influence various aspects of psychological well-being and may indirectly affect mental health by influencing attitudes, behaviors, and life satisfaction [31]. Research shows that personal values also play a crucial role in resilience [32]. While external factors such as excessive workload, inadequate psychological safety, and workplace bullying need to be addressed through targeted interventions to promote a resilient and effective healthcare workforce [33], the significant mental health challenges faced by medical staff highlight the need to consider whether medical students themselves can be educated to adopt healthier behaviors or perhaps even selection that prioritizes mental health is more effective. This study examines the relationship between personality traits, values, demographic characteristics, and attitudes toward professionalism to determine whether interventions throughout the educational process could promote healthier and more functional medical students or whether prioritizing mental health in selection criteria could better ensure their well-being.

## 2. Materials and Methods

### 2.1. Participants and Procedure

This was a six-year national study among medical students at the two Slovenian medical faculties Ljubljana and Maribor. All medical students enrolled in the fourth and sixth (final) year of study at both faculties were invited to participate. The data collection was carried out in the academic years 2015/6, 2017/18 and 2019/20. The three consecutive data collections were designed to provide sufficient data to identify the most robust associations between personality, values, and traits known to be related to professional behavior toward patients and also to the well-being of acting professionals.

Students were briefed on the research and the instrument and took approximately 45 min to complete the questionnaire. Data collection took place during tutorials to maximize participation, as the length of the questionnaire might otherwise discourage students from participating in their free time. Participation was voluntary. Students gave their informed consent by completing and submitting the psychodiagnostic toolkit. As the pool was very large, we did not require a separate informed consent form. Participants were informed at the beginning that they could terminate their participation at any time or that they had given their consent to participate in the study by submitting the completed questionnaires.

### 2.2. Instruments

The most important personality traits were measured using the Big Five Questionnaire (BFQ) developed by Caprara, Barbaranelli, and Borgogni, which measures five personality traits (extraversion, agreeableness, conscientiousness, neuroticism, and openness) using 132 items with five-point Likert ratings (5 = applies very much to me; 1 = does not apply to me at all). The Slovenian version of the BFQ has shown adequate reliability and validity [34,35]

Values were assessed using the Scale of Personal Values (SPV), a psychodiagnostic instrument developed to assess the motivational structure of adolescents and adults by categorizing 24 values (partner/love; children; hobbies/leisure; personal safety/health; moral/ethical principles; sports; friends; career/work; sociability; self-esteem; freedom/independence; new experiences; sexuality; knowledge/wisdom; food/drink; parents/home; rest; beauty/art; comfort/pleasure; creativity; property/money; faith/God; power/influence; and prestige/fame) into seven levels of importance. The SPV is reliable, with a test–retest reliability between 0.46 and 0.95, and effectively identifies a person’s five to ten most important values [36]. When analyzing personal values in relation to attitudes toward professionalism, we formed four higher order values: Active (community, new experiences, friends, self-esteem, freedom/independence, sporting activities, prestige/fame); Passive (food/drink, power/influence, personal security/health, children, partner/love, rest, parents/home); Material (hobbies/leisure, property/money, sexuality, comfort/pleasure); and Spiritual (beauty/art, morals/ethics, profession/work, creativity, faith/God, knowledge/wisdom).

Attitudes toward professionalism were assessed using the Professionalism Attitude Scale (PAS). The PAS, a 22-item instrument developed by Klemenc-Ketiš and Vrecko in Slovenia in 2014, has been shown to be a reliable and valid measure of medical students’ attitudes toward medical students [37].

All instruments used had appropriate metric properties and have been described in detail elsewhere [10].

### 2.3. Data Analysis

Sample data were presented as frequencies and percentages or as means and SDs. Fisher’s exact test was used to examine the demographic differences between the two Slovenian medical schools. Hierarchical linear regression analysis was used to determine the factors associated with medical students’ attitudes toward professionalism. The calculation included the beta coefficient, *t*-value, and *p*-value. For each step of the analysis, we also reported R^2^ along with the R^2^ change (ΔR^2^).

When analyzing the personal values related to the attitude toward professionalism, a principal axis factoring as a method for extracting factors from the original correlation matrix with squared multiple correlation coefficients in the diagonal as the first estimate of commonality was performed with varimax rotation and eigenvalues >1. Varimax rotation was an important second step in factor analysis and principal component analysis. Factor rotation, including varimax rotation, transformed the original factors into new factors that are easier to interpret. Items with a factor loading below 0.4 were suppressed [38]. The resulting two-factor solution explained 57.7% of the original variance.

Statistical analysis was performed using IBM SPSS 23.0 software (IBM Corp., Armonk, NY, USA). *p* < 0.05 was marked as statistically significant.

## 3. Results

A total of 996 people took part in the study, 65.5% (652) of them from the Faculty of Medicine in Ljubljana and 34.5% (334) from the Faculty of Medicine in Maribor. This ratio reflects the relative size of the faculties, as about twice as many students are enrolled at the faculty in Ljubljana than at the faculty in Maribor. In the 2015/16 academic year, for example, 206 out of 416 students at the Faculty of Medicine in Ljubljana took part (49.5% response rate) and 117 out of 158 at the Faculty of Medicine in Maribor (74.1% response rate). In total, 323 out of 576 invited students participated in the study (response rate of 56.1%).

The demographic characteristics of the participants and personality traits by year of data collection are shown in Table 1. Because Fisher’s exact test revealed no differences between the students from the two faculties, we analyzed the remaining results together, representing the first national study of attitudes toward professionalism. More detailed descriptions of demographic data and personality traits based on the year of data collection can be found in the Supplemental Materials (Supplemental Materials, Tables S1–S7).

After principal axis factoring (Table 2), the PAS total score was associated with the superordinate groups of Passive (food/drink, power/influence, personal safety/health, children/partner/love, rest, parents/home) and Material (hobbies/leisure, property/money, sexuality, comfort/pleasure) value orientations. In the multivariate analysis (Table 3), we used only the superordinate groups of passive and material value orientation to define the associations with attitudes toward professionalism.

In the multivariate modeling, there was only a negative association with material value orientation in 2015/16 and 2019/20, but not in the 2017/18 data collection. Female gender and acceptance were independently associated with a higher score in the PAS after Step 3 in all data collections. Conscientiousness was associated with a higher PAS score in the first two data collections, but not in the third. Rural environment was negatively associated with a higher PAS score in the 2019/20 group, but not in the first two groups. In Step 1 of the hierarchical linear regression modeling, 13% of the variance was explained; in Step 2, personal values explained another 8% and basic personality traits in Step 3 contributed another 10% to a total of 31% of the explained variance in attitudes toward professionalism (Table 3).

Overall, the PAS scores remained stable across the three repeated data collections, and the most stable relationship was between female gender and agreeableness, with higher PAS scores observed in all three years. A positive relationship between higher PAS scores and conscientiousness and an inverse relationship between higher PAS scores and material value orientation was demonstrated for two of the three years (Table 3).

## 4. Discussion

To our knowledge, this is the first study to examine personality profiles and attitudes toward professionalism with implications for future performance and well-being of participating medical students at a national level. The aim of this study was to identify the factors that are reliably associated with more pronounced attitudes toward professionalism and to facilitate discussion on the criteria for admission to medical school in Slovenia.

### 4.1. Is There a Typical Study Participant and What Can We Expect from Them?

Based on the BFQ scores, the “typical” student in our study can be described as follows (Table 1): not very energetic or dominant (weak extroversion), fairly altruistic and cooperative (moderate agreeableness), fairly emotionally stable (moderate neuroticism), reasonably responsible (moderate conscientiousness), and not very open to new experiences (moderate openness overall, but weak in the openness to experience subcategory). Slight variations were observed between the 2015/16, 2017/18 and 2019/20 cohorts, such as higher extraversion in 2015/16 (moderate extroversion) and higher neuroticism in 2019/20 (which falls under the reverse descriptor “not very emotionally stable”), but overall, the personality traits remained the same (Table 1).

In other words, a “typical medical student” in Slovenia is cooperative, empathetic, and responsible, yet shy, anxious, and with a conventional way of thinking compared to their classmates. Given the known links between personality traits and mental health, these results could be problematic when it comes to students’ resilience in their future roles as doctors.

The Big Five are a strong predictor of mental health [17], are related to resilience (with Neuroticism showing a negative association) [16], and each contributes to mental health in different ways. Extraversion, on which students in our study scored repeatedly and therefore reliably poorly, is associated with higher levels of subjective well-being, positive affect, and social support, which contribute to better mental health outcomes [20]. Openness to experience, on which participants scored moderately, is associated with better mental health [21]. Agreeableness, on which Slovenian medical students scored moderately, is also clearly associated with positive mental health in the literature [22]. Conscientiousness, on which participants also scored moderately, predicts better academic and professional performance [23]. Neuroticism, on which students also scored moderately, is strongly associated with a higher prevalence of mood disorders such as depression and anxiety and is a vulnerability factor for mental health problems [24].

As for the differences between the students in the study and the general population, all scores except for conscientiousness were below the Slovenian norm for this age group, as shown previously [39]. One explanation for this could lie in the selection process for admission to medical school in Slovenia. Selection is strictly based on academic success throughout school and in the final exam, without an additional test in medical school. Studies show that conscientiousness is the strongest non-cognitive predictor of academic success in adolescents [40]. Moreover, a low score on the openness scale might even be an advantage for academic performance in a rigorous high school environment, as has been shown for dental school [41]. Thus, medical schools may unintentionally select the more conscientious and less open-minded students, which could ultimately have a negative impact on their mental and physical well-being.

**Figure 1:**
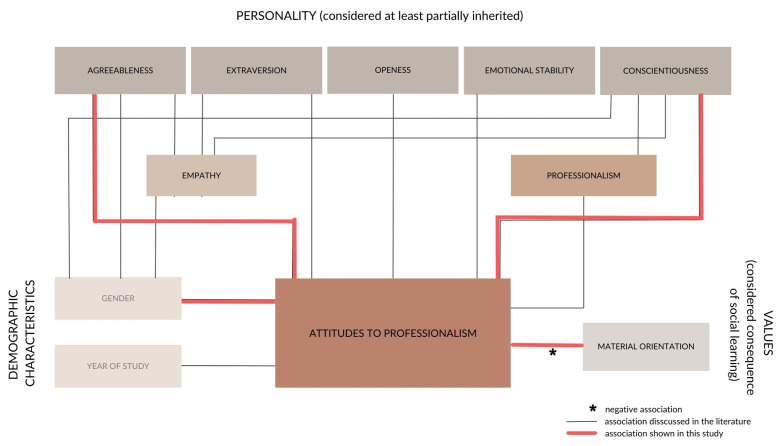
Relationships between the variables in this study. All variables (with the exception of year of study) have been previously linked to mental health, which has been omitted from the figure for clarity.

Another aim of the study was to assess the relationship between personality traits, values, and demographic characteristics and attitudes toward professionalism (Table 3, Figure 1). The PAS is commonly used to assess attitudes toward professionalism in various professions, including healthcare, and professional beliefs are closely related to the well-being of physicians and patients [8,12,13].

### 4.2. The Values of Future Doctors in Slovenia: Are They a Good Reference for Healthy Professionals?

For values, which we considered a by-product of early social learning, the only association was an inverse relationship between materialistic value orientation and PAS score (Table 3, Figure 1), suggesting that students with high conceptions of the medical profession are motivated by factors other than material gain. A meta-analysis revealed a significant negative relationship between individuals’ materialistic orientation and their personal well-being [31].

### 4.3. Is the Importance of Personality for Professional Performance and Personal Well-Being Exaggerated in Medicine?

Of the personality traits that we hypothesized to be at least partially heritable, there were the strongest correlations with Agreeableness and Conscientiousness (Table 3, Figure 1), which have been strongly associated with professionalism in healthcare professionals [25,26] and which the literature suggests are associated with better mental health and well-being in many domains.

In particular, conscientiousness is associated with self-discipline, thoroughness, persistence, and motivation for goal-directed behavior, higher grade point average and academic performance [23], career success, and also professional behavior [27]. While it is unlikely that conscientiousness encompasses all professionalism, significant positive correlations were found between Clinical Conscientiousness Index scores and professionalism reported by others [29], so conscientiousness can be considered a proxy for professionalism. Higher levels of conscientiousness are associated with greater psychological well-being and stress resilience [16,17], as well as better physical health and longer life expectancy [28].

Agreeableness refers to friendliness, social competence, altruistic, cooperative behavior, and pro-social behavior [42] and also affects psychological well-being and interpersonal relationships [22].

Previous research has repeatedly shown that conscientiousness and especially agreeableness are the most important predictors of sympathy and empathy [42,43,44,45]. Empathy is a central attribute of professionalism in clinical practice [46] and consists of the ability to understand and share the emotional states of other people. The former is a cognitive experience that is beneficial and related to better patient outcomes, higher career satisfaction, and protection from burnout. The latter is an emotional experience that can impair objectivity and lead to exhaustion, compassion fatigue, burnout, and vicarious traumatization in a medical setting [47].

Other studies have also found correlations between empathy and other personality traits such as extraversion [48] and openness to experience [46] (Figure 1). Of course, empathy requires a certain predisposition to sensitivity and openness to the views, experiences and perspectives of others. However, openness to experience was significantly lower in the medical students in this study (Table 1) than in the general population, but as mentioned earlier, this could be a by-product of medical students being selected for their academic performance.

### 4.4. Female Doctors: Can They Act More Professionally and Stay Healthier?

In terms of demographic characteristics, the female gender was significantly and stably associated with a higher score on the Attitude Toward Professionalism Scale (Table 3, Figure 1).

The literature indicates that, in general and across cultures, women score significantly higher than men on Conscientiousness and Agreeableness, as well as Neuroticism and Openness to Emotions, while men score higher on Openness to Ideas and the trait associated with Extraversion, Assertiveness [29]. The fact that the majority of students in the study was female may be an additional factor in the association of Conscientiousness and Agreeableness with higher PAS scores (Table 3). It is also noteworthy that females tended to score higher on empathy [45] and were better protected from the erosion of empathy that is otherwise prevalent in the medical field [47].

Whether the gender differences in personality [29] and empathy are due to nature (innate biological traits) or nurture (the role of socialization and gender roles imposed by society) remains controversial, as the influence of one and the other is difficult to separate practically and impartially. Indeed, the Western construct of femininity requires women to develop the notions of kindness, emotional empathy, caring, and self-sacrifice associated with empathy [43]. Interestingly, studies in Asia and Western areas with a significant Asian population have found similar scores in both genders, which may be due to differences in upbringing, cultural values, and gender roles in different societies [45].

### 4.5. Gathering Knowledge about Professionalism or Training for More Professionalism: Where Is Medical Education in Slovenia Heading?

Remarkably, the PAS scores remained stable across the three data collections and no significant differences were found between Year 4 and Year 6 students (Table 3, Figure 1). This is in contrast to previous studies, which have found an increase in negative views of primary care, lower perceptions of the status and income of physicians, and a decrease in idealism among older students [10,11]. In addition to a decline in idealism, studies have also found a decline in empathy, another important component of professionalism [49], which is particularly evident in the third year of medical school and can be attributed to the transition between preclinical and clinical years in the four-year programs addressed in the foreign studies [47].

Since the program examined in this study is a six-year program with the clinical years beginning in the fourth year, this is when we would expect to see a change in attitudes. There could be many factors as to why our results are not in line with our expectations. It is possible that we might see a change in attitudes if we were to observe students longer and assess them as they enter residency, when a further loss of empathy might be expected [47]. In recent years, medical schools have introduced a curriculum in which students have some contact with patients in the first year. In the third year, at least one day per week is devoted to interaction with patients, in pairs or small groups, under the guidance of an assigned mentor. On the one hand, therefore, it is possible that the de facto transition to the clinical years occurs in the third year of medical school rather than the formal fourth year and that students were simply not surveyed early enough to recognize the change in environment.

On the other hand, it is possible that the curriculum prevented a change in attitude in the first place, as the early patient contact and small group discussions led to a better understanding and embodiment of professionalism [50,51]. However, there are other factors to consider. The majority of students in the study were women (Table 1), who were more likely to resist a loss of empathy [47]. It should also be noted that studies of Japanese [52] and Korean [30] medical students found no decline in empathy, which was attributed to cultural differences, the role of the doctor in society and differences in the healthcare system—for some of these factors, Slovenia may be more closely related to Japan and Korea than to the USA, where many of the other studies took place. One explanation worth considering could be that the participants in this study experienced a more positive attitude from their mentors and had fewer negative experiences in their educational journey than students in other cultures. It is therefore possible that there are interventions that could help to strengthen attitudes toward professionalism throughout the educational process and promote resilience to psychological stressors.

### 4.6. Different Weighting of the Selection Criteria: Which Characteristics Could Ensure Better Well-Being in the Future Profession?

There is a need to refine the selection criteria that emphasize mental health to ensure the well-being of future physicians by selecting medical students who are most likely to produce healthier and more functional physicians, possibly with an emphasis on personality and values.

Medical schools have a duty to ensure that successful applicants are ultimately able to practice. That said, late adolescence to early adulthood is a turbulent and developmentally challenging time. While the Big Five are generally considered very stable in adulthood [15], the personality continues to develop during adolescence and the period between late adolescence and early adulthood, when most students choose to study medicine, is a formative time, in which the Big Five are still malleable and immature [53].

While value theorists believe that values are generally stable across situations and time, they also assume that value priorities change throughout life as people adapt to important experiences and changing circumstances [54]. Therefore, a selection criterion might be both inaccurate and unfair, which should be taken into account in future discussions about medical schools in Slovenia.

### 4.7. Strengths and Contributions

This study is characterized by its national scope. It includes medical students from both Slovenian medical schools and provides a comprehensive overview of professionalism and personality traits on a national level over a six-year period. This approach, combined with a relatively high response rate (56.1% overall) and well-established and validated instruments, provides a reliable, robust data set and enables the examination of changes and stability in attitudes toward professionalism over time, with a broad, reliable, and representative national perspective on the topic, with potential implications for medical education and policy.

The study provides a detailed analysis of characteristics that influence attitudes to professionalism and mental health, including personality traits, values, and gender, which could inform the development of medical student training and selection procedures, as well as targeted support and mentoring strategies. The findings suggest that the current medical curriculum in Slovenia can promote positive attitudes toward professionalism, offering insights for curriculum development and possible interventions abroad as well. By identifying important associations and trends, this groundbreaking study on professionalism in Slovenian medical students lays the foundation for further research on how personality and values influence the professional behavior and well-being of medical professionals, both in Slovenia and internationally.

### 4.8. Limitations and Future Research Directions

The study included a national sample of medical students from two Slovenian medical schools. However, the response rates varied between the two faculties (e.g., 49.5% in Ljubljana vs. 74.1% in Maribor in 2015/16), which means that the different response rates could lead to biases that affect the generalizability of the results. Participation was voluntary, which might lead to self-selection bias, as students who chose to participate may differ significantly from those who did not. The self-report measures used in the study, such as the BFQ and the Professionalism Attitude Scale, may lead to response bias as participants may give socially desirable responses, which could skew the results. However, it should be noted that the instruments in this study use several strategies to mitigate these biases, such as reverse-scored items, social desirability scales, and redundancy to improve reliability. Slovenian versions of the questionnaires were used to mitigate biases that may arise from cultural differences in the perception or evaluation of traits. However, the assessment of personality and values requires self-reporting, which inherently carries the risk of certain biases that are unlikely to be effectively eliminated in future research.

In the study, a hierarchical linear regression analysis was used to assess associations. However, this approach does not account for causality or the potential effects of unmeasured confounding variables. Longitudinal studies following physicians at different stages of their medical education (including premedical training, medical school, and postgraduate training) to examine how attitudes, values, and personality traits develop in their professional practice could provide deeper insights into the directionality of these relationships and the long-term effects of medical education on professionalism.

The factor analysis used to create higher order values may have oversimplified or overlooked some nuances in value orientations. The suppression of items with factor loadings below 0.4 may have excluded important variables. Demographic characteristics, personal values, and personality traits explained 13%, 8%, and 10% of the variance, respectively, accounting for 31% of the total variance, which means that many cofounding factors may remain unexplored. For example, a study of nursing students during the COVID-19 pandemic examined how emotional regulation and higher levels of psychological capital (which includes elements such as resilience, optimism, and self-efficacy) mitigated negative emotions and risks during a pandemic and increased their commitment to the nursing profession [14]. These are just a few of the many confounding characteristics that could influence the complex interplay of personality traits, values, attitudes toward professionalism, and mental health.

This study did not use objective measures of mental health, such as standardized mental health questionnaires, frequency of mental health diagnoses, or statistics on student use of services, attendance and participation logs, or performance metrics. However, future research could use such indicators to confirm a more direct influence of professionalism, personality traits, and values on mental health, resilience, and burnout rates. Specific training programs could be developed and evaluated to specifically investigate the effectiveness of pedagogical interventions to promote professionalism and personal values. Qualitative research could also provide deeper insights into the experiences and perceptions of medical students and professionals.

## 5. Conclusions

This is the first study to look at the personality profiles of medical students and their attitudes toward professionalism at a national level. Personality traits, values, and attitudes toward professionalism were fairly consistent over the years. Students’ personality traits were stable but below the norm for the population (with the exception of conscientiousness), which could be a by-product of either medical school selection or adolescent development, in which case mentoring as a role model could be beneficial to students.

The PAS scores also remained stable, suggesting that the current curriculum is likely to prevent deterioration in students’ appropriate attitudes toward professionalism. Women consistently showed more positive attitudes toward professionalism. Associations were also found with agreeableness, conscientiousness, and inversely with material value orientation.

All of the factors described are closely related to the mental health of doctors. As healthcare professionals are increasingly burdened with stress and burnout due to the high demands of their work, it is becoming increasingly important to take care of their mental health.

We hope that this study can lead to a better evaluation of the medical student selection process, training requirements, and medical schools, and improve and maintain the well-being of physicians; however, further research is needed in this area.

## Figures and Tables

**Table 1:** Participant demographics and personality traits by year of data collection.

	Data Collection 2015/16 [n = 323]		Data Collection 2017/18 [n = 339]		Data Collection 2019/20 [n = 334]	
	N	%	N	%	N	%
**Faculty of Medicine**						
Ljubljana	206	63.8	221	65.2	225	67.4
Maribor	117	36.2	118	34.8	109	32.6
**Gender**						
Female	222	68.7	238	70.2	225	67.4
Male	101	31.3	101	29.8	109	32.6
**Year of study**						
4th	183	56.7	178	52.5	180	56.9
6th	140	43.3	161	47.8	154	46.1
	**M**	**SD**	**M**	**SD**	**M**	**SD**
Extroversion	45.4	9.2	44.6	9.4	43.5	9.0
Agreeableness	47.4	9.9	47.5	9.7	46.8	10.9
Conscientiousness	50.4	9.3	49.5	9.4	49.7	9.9
Emotional stability	46.7	9.3	45.5	9.4	44.9	10.0
Openness	46.1	9.1	45.5	9.2	46.8	9.3
L-score	49.2	8.2	47.3	8.0	46.6	8.2

**Table 2:** Results of principal axis factorization with Varimax rotation. The 24 values analyzed and the total PAS score. The 24 values were divided into four higher order values: Active, Passive, Material and Spiritual (described in more detail in Data Analysis). Significant associations are shaded, with F1 representing orientation toward passive values and F2 representing orientation toward material values.

	F1	F2
V1: Social gathering	0.033	0.096
V2: Hobbies/free time	−0.003	0.503
V3: Food/drink	0.441	0.232
V4: Property/money	−0.013	0.613
V5: Beauty/art	0.172	−0.087
V6: Power/influence	0.538	0.113
V7: Moral, ethical principles	−0.005	−0.166
V8: New experiences	0.228	0.025
V9: Personal safety/health	0.517	0.104
V10: Children	0.725	−0.145
V11: Partner/love	0.792	−0.031
V12: Rest	0.443	0.148
V13: Profession/work	−0.002	−0.079
V14: Friends	0.024	0.136
V15: Self-esteem	0.116	0.052
V16: Sexuality	0.012	0.448
V17: Parents/home	0.560	0.043
V18: Freedom/independence	0.112	−0.070
V19: Sport activity	−0.012	0.098
V20: Comfort/pleasure	−0.059	0.671
V21: Prestige/fame	0.236	0.078
V22: Creativity	0.227	−0.283
V23: Faith/God	−0.189	−0.214
V24: Knowledge/wisdom	0.264	−0.193

**Table 3:** Multivariate analysis of the relationship between personality traits, values and demographic characteristics with attitudes toward professionalism. Significant associations are shaded.

	Data Collection 15/16			Data Collection 17/18			Data Collection 19/20		
	beta	*t*	*p*	beta	*t*	*p*	beta	*t*	*p*
Demographic Characteristics									
Year of Study (6th vs. 4th)	−0.09	−1.59	0.112	−0.01	−0.15	0.883	0.07	1.46	0.144
Gender (Male vs. Female)	−0.30	−5.30	<0.001	−0.18	−3.74	<0.001	−0.19	−4.10	<0.001
Environment (Rural vs. Urban)	0.01	0.12	0.908	−0.03	−0.58	0.560	−0.09	−2.02	0.044
Siblings (Yes vs. No)	−0.01	−0.22	0.823	−0.02	−0.41	0.682	−0.02	−0.34	0.732
Father’s Level of Education	0.07	1.09	0.278	0.02	0.37	0.710	−0.01	−0.18	0.857
Mother’s Level of Education	0.08	1.40	0.133	−0.08	−1.24	0.217	−0.07	−1.54	0.126
R^2^	0.131			0.080			0.103		
Demographic Characteristics									
Year of Study (6th vs. 4th)	−0.08	−1.39	0.167	−0.01	−0.13	0.899	0.07	1.62	0.105
Gender (Male vs. Female)	−0.27	−4.51	<0.001	−0.18	−3.63	<0.001	−0.15	−3.35	0.001
Environment (Rural vs. Urban)	−0.01	−0.12	0.901	−0.03	−0.60	0.549	−0.11	−2.39	0.017
Siblings (Yes vs. No)	−0.02	−0.28	0.779	−0.02	−0.42	0.672	−0.01	−0.26	0.798
Father’s Level of Education	0.07	1.10	0.274	0.03	0.48	0.630	−0.02	−0.28	0.781
Mother’s Level of Education	0.07	1.22	0.227	−0.08	−1.27	0.203	−0.07	−1.32	0.121
Value orientation									
Passive	0.04	0.73	0.468	0.03	0.70	0.481	0.07	1.57	0.117
Material	−0.16	−2.77	0.006	0.03	0.67	0.504	−0.13	−2.97	0.003
R^2^ (∆R^2^)	0.156 (+0.025)			0.092 (+0.012)			0.126 (+0.023)		
Demographic Characteristics									
Year of Study (6th vs. 4th)	−0.08	−1.45	0.149	0.00	0.08	0.938	0.05	1.22	0.225
Gender (Male vs. Female)	−0.27	−4.70	<0.001	−0.16	−3.44	0.001	−0.13	−2.94	0.003
Environment (Rural vs. Urban)	−0.03	−0.45	0.653	−0.02	−0.51	0.611	−0.07	−1.59	0.147
Siblings (Yes vs. No)	−0.01	−0.12	0.907	−0.03	−0.58	0.564	−0.01	−0.29	0.770
Father’s Level of Education	0.09	1.47	0.142	0.03	0.48	0.634	−0.01	−0.27	0.784
Mother’s Level of Education	0.05	1.04	0.253	−0.06	−1.09	0.277	−0.06	−1.24	0.216
Value orientation									
Passive	0.04	0.76	0.450	0.02	0.42	0.674	0.08	1.77	0.078
Material	−0.10	−1.67	0.096	0.08	1.76	0.079	−0.08	−1.70	0.089
BFQ dimension (*t* value)									
Extroversion	−0.09	−1.62	0.107	0.03	0.68	0.496	−0.04	−0.92	0.360
Agreeableness	0.30	5.22	<0.001	0.24	4.90	<0.001	0.28	5.96	<0.001
Conscientiousness	0.11	2.47	0.025	0.12	2.60	0.010	0.05	1.28	0.213
Emotional stability	0.05	0.94	0.349	0.00	−0.10	0.922	−0.04	−0.84	0.403
Openness	−0.03	−0.44	0.659	−0.04	−0.69	0.489	0.03	0.55	0.579
R^2^ (∆R^2^)	0.253 (+0.097)			0.187 (+0.095)			0.214 (+0.088)		

## Data Availability

Data sets used and/or analyzed in the current study are available upon request from the corresponding author.

## Supplementary Materials

The following supporting information can be downloaded at: https://www.mdpi.com/article/10.3390/healthcare12171732/s1, More detailed descriptions of demographic data and personality traits based on the year of data collection, Tables S1–S7. Table S1. The demographic data of participants by faculty and data collection year. Table S2. The demographic data of participants by faculty for the 2015/16 data collection year. Table S3. The demographic data of participants by faculty for the 2017/18 data collection year. Table S4. The demographic data of participants by faculty for the 2019/20 data collection year. Table S5. Big Five Values: 2015/16 data collection raw values compared to the reference sample. Table S6. Big Five Values: 2017/18 data collection raw values compared to the reference sample. Table S7. Big Five Values: 2019/20 data collection raw values compared to the reference sample.

## References

[1] Harvey S.B., Epstein R.M., Glozier N., Petrie K., Strudwick J., Gayed A., Dean K., Henderson M. (2021). Mental illness and suicide among physicians. Lancet.

[2] Dyrbye L.N., West C.P., Satele D., Boone S., Tan L., Sloan J., Shanafelt T.D. (2014). Burnout among U.S. medical students, residents, and early career physicians relative to the general U.S. population. Acad. Med..

[3] Rotenstein L.S., Ramos M.A., Torre M., Segal J., Peluso M.J., Guille C., Sen S., Mata D.A. (2016). Prevalence of depression, depressive symptoms, and suicidal ideation among medical students: A systematic review and meta-analysis. JAMA-J. Am. Med. Assoc..

[4] Heinen I., Bullinger M., Kocalevent R.D. (2017). Perceived stress in first year medical students—Associations with personal resources and emotional distress. BMC Med. Educ..

[5] Shemtob L., Good L., Ferris M., Asanati K., Majeed A. (2022). Supporting healthcare workers with work-related stress. BMJ.

[6] Spoorthy M.S., Pratapa S.K., Mahant S. (2020). Mental health problems faced by healthcare workers due to the COVID-19 pandemic–A review. Asian J. Psychiatry.

[7] Cyr S., Marcil M.J., Houchi C., Marin M.F., Rosa C., Tardif J.C., Guay S., Guertin M.-C., Genest C., Forest J. (2022). Evolution of burnout and psychological distress in healthcare workers during the COVID-19 pandemic: A 1-year observational study. BMC Psychiatry.

[8] Aiken L.H., Lasater K.B., Sloane D.M., Pogue C.A., Fitzpatrick Rosenbaum K.E., Muir K.J., McHugh M.D., US Clinician Wellbeing Study Consortium (2023). Physician and nurse well-being and preferred interventions to address burnout in hospital practice: Factors associated with turnover, outcomes, and patient safety. JAMA Health Forum.

[9] Papadakis M.A., Paauw D.S., Hafferty F.W., Shapiro J., Byyny R.L. (2012). Perspective: The education community must develop best practices informed by evidence-based research to remediate lapses of professionalism. Acad. Med..

[10] Selic P., Cerne A., Klemenc-Ketis Z., Petek D., Svab I. (2019). Attitudes toward professionalism in medical students and its associations with personal characteristics and values: What actually makes a difference?. Adv. Med. Educ. Pract..

[11] Johnston J.L., Cupples M.E., McGlade K.J., Steele K. (2011). Medical students’ attitudes to professionalism: An opportunity for the GP tutor?. Educ. Prim. Care.

[12] Dyrbye L.N., Massie F.S., Eacker A., Harper W., Power D., Durning S.J., Thomas M.R., Moutier C., Satele D., Sloan J. (2010). Relationship between burnout and professional conduct and attitudes among US medical students. JAMA.

[13] Dyrbye L.N., Harper W., Moutier C., Durning S.J., Power D.V., Massie F.S., Eacker A., Thomas M.R., Satele D., Sloan J.A. (2012). A multi-institutional study exploring the impact of positive mental health on medical students’ professionalism in an era of high burnout. Acad. Med..

[14] Li J., Huang C., Yang Y., Liu J., Lin X., Pan J. (2023). How nursing students’ risk perception affected their professional commitment during the COVID-19 pandemic: The mediating effects of negative emotions and moderating effects of psychological capital. Humanit. Soc. Sci. Commun..

[15] Babcock S.E., Wilson C.A. (2020). Big five model of personality. The Wiley Encyclopedia of Personality and Individual Differences, Personality Processes and Individuals Differences.

[16] Oshio A., Taku K., Hirano M., Saeed G. (2018). Resilience and Big Five personality traits: A meta-analysis. Pers. Individ. Differ..

[17] Strickhouser J.E., Zell E., Krizan Z. (2017). Does personality predict health and well-being? A metasynthesis. Health Psychol..

[18] Anglim J., Horwood S., Smillie L.D., Marrero R.J., Wood J.K. (2020). Predicting psychological and subjective well-being from personality: A meta-analysis. Psychol. Bull..

[19] Turiano N.A., Whiteman S.D., Hampson S.E., Roberts B.W., Mroczek D.K. (2012). Personality and substance use in midlife: Conscientiousness as a moderator and the effects of trait change. J. Res. Pers..

[20] Steel P., Schmidt J., Shultz J. (2008). Refining the Relationship Between Personality and Subjective Well-Being. Psychol. Bull..

[21] Kang W., Steffens F., Pineda S., Widuch K., Malvaso A. (2023). Personality traits and dimensions of mental health. Sci. Rep..

[22] Lamers S.M.A., Westerhof G.J., Kovács V., Bohlmeijer E.T. (2012). Differential relationships in the association of the Big Five personality traits with positive mental health and psychopathology. J. Res. Pers..

[23] Vedel A. (2014). The Big Five and tertiary academic performance: A systematic review and meta-analysis. Pers. Individ. Differ..

[24] Widiger T.A., Oltmanns J.R. (2017). Neuroticism is a fundamental domain of personality with enormous public health implications. World Psychiatry.

[25] Qasemi L., Behroozi M. (2015). Survey of Personality Traits [Based on Big Five] In Professional Ethics’s Growth In Medical Sciences University of Bushehr. Iran’s Aspect. Procedia Soc. Behav. Sci..

[26] Masmouei B., Bazvand H., Harorani M., Bazrafshan M.R., Karami Z., Jokar M. (2020). Relationship Between Personality Traits and Nursing Professionalism. J. Client-Cent. Nurs. Care.

[27] McLachlan J.C., Finn G., Macnaughton J. (2009). The conscientiousness index: A novel tool to explore students’ professionalism. Acad. Med..

[28] Kern M.L., Friedman H.S., Martin L.R., Reynolds C.A., Luong G. (2009). Conscientiousness, career success, and longevity: A lifespan analysis. Ann. Behav. Med..

[29] Kelly M., O’Flynn S., McLachlan J., Sawdon M.A. (2012). The clinical conscientiousness index: A valid tool for exploring professionalism in the clinical undergraduate setting. Acad. Med..

[30] Roh M.-S., Hahm B.-J., Lee D.H., Suh D.H. (2010). Evaluation of empathy among Korean medical students: A cross-sectional study using the Korean version of the Jefferson scale of physician empathy. Teach. Learn. Med..

[31] Dittmar H., Bond R., Hurst M., Kasser T. (2014). The relationship between materialism and personal well-being: A meta-analysis. J. Pers. Soc. Psychol..

[32] Athota V.S., Budhwar P., Malik A. (2020). Influence of personality traits and moral values on employee well-being, resilience, and performance: A cross-national study. Appl. Psychol..

[33] Brand S.L., Coon J.T., Fleming L.E., Carroll L., Bethel A., Wyatt K. (2017). Whole-system approaches to improving the health and well-being of healthcare workers: A systematic review. PLoS ONE.

[34] Caprara G.V., Barbaranelli C., Borgogni L., Perugini M. (1993). The “big five questionnaire”: A new questionnaire to assess the five factor model. Pers. Individ. Differ..

[35] Caprara G.V., Barbaranelli C., Borgogni L., Bucik V., Boben D., Hruševar-Bobek B., Zupančič M., Horvat M. (2012). Model “Velikih Pet”: Pripomočki za Merjenje Strukture Osebnosti: Priročnik.

[36] Pogavcnik V., Musek J. (2002). Lestvica osebnih vrednot-LOV: Prirocnik.

[37] Klemenc-Ketis Z., Vrecko H. (2014). Development and validation of a professionalism assessment scale for medical students. Int. J. Med. Educ..

[38] Hair J.F., Black W.C., Babin B.J., Anderson R.E. (2010). Multivariate Data Analysis. Vectors.

[39] Meit S.S., Borges N.J., Early L.A. (2007). Personality Profiles of Incoming Male and Female Medical Students: Results of a Multi-Site 9-Year Study. Med. Educ. Online.

[40] Dumfart B., Neubauer A.C. (2016). Conscientiousness is the most powerful noncognitive predictor of school achievement in adolescents. J. Individ. Differ..

[41] Smithers S., Catano V.M., Cunningham D.P. (2004). What Predicts Performance in Canadian Dental Schools?. J. Dent. Educ..

[42] Habashi M.M., Graziano W.G., Hoover A.E. (2016). Searching for the prosocial personality. Pers. Soc. Psychol. Bull..

[43] Melchers M.C., Li M., Haas B.W., Reuter M., Bischoff L., Montag C. (2016). Similar Personality Patterns Are Associated with Empathy in Four Different Countries. Front. Psychol..

[44] Romosan R.S., Dehelean L., Enatescu V.R., Bredicean A.C., Papava I., Giurgi-Oncu C., Romosan A.-M. (2018). Profiling undergraduate students from a Romanian medical university. Neuropsychiatr. Dis. Treat..

[45] O’tuathaigh C.M.P., Idris A.N., Duggan E., Costa P., Costa M.J. (2019). Medical students’ empathy and attitudes towards professionalism: Relationship with personality, specialty preference and medical programme. PLoS ONE.

[46] Magalhães E., Costa P., Costa M.J. (2012). Empathy of medical students and personality: Evidence from the Five-Factor Model. Med. Teach..

[47] Hojat M., Vergare M.J., Maxwell K., Brainard G., Herrine S.K., Isenberg G.A., Veloski J., Gonnella J.S. (2009). The Devil is in the Third Year: A Longitudinal Study of Erosion of Empathy in Medical School. Acad. Med..

[48] Nettle D. (2007). Empathizing and systemizing: What are they, and what do they contribute to our understanding of psychological sex differences?. Br. J. Psychol..

[49] Magalhães E., Salgueira A.P., Costa P., Costa M.J. (2011). Empathy in senior year and first year medical students: A cross-sectional study. BMC Med. Educ..

[50] Monrouxe L.V., Rees C.E., Hu W. (2011). Differences in medical students’ explicit discourses of professionalism: Acting, representing, becoming. Med. Educ..

[51] Birden H., Glass N., Wilson I., Harrison M., Usherwood T., Nass D. (2013). Teaching professionalism in medical education: A Best Evidence Medical Education (BEME) systematic review. BEME Guide No. 25. Med. Teach..

[52] Kataoka H.U., Koide N., Ochi K., Hojat M., Gonnella J.S. (2009). Measurement of empathy among Japanese medical students: Psychometrics and score differences by gender and level of medical education. Acad. Med..

[53] Bleidorn W., Schwaba T., Zheng A., Hopwood C.J., Sosa S.S., Roberts B.W., Briley D.A. (2022). Personality stability and change: A meta-analysis of longitudinal studies. Psychol. Bull..

[54] Vecchione M., Schwartz S., Alessandri G., Döring A.K., Castellani V., Caprara M.G. (2016). Stability and change of basic personal values in early adulthood: An 8-year longitudinal study. J. Res. Pers..

