# Supplementary material for: Exploring Personality Traits, Values, and Attitudes toward Professionalism: Implications for the Promotion of Mental Health and Functioning in Medical Students
Source: Healthcare (Basel). 2024 Aug 30;12(17):1732. doi: 10.3390/healthcare12171732 (PMC11394833; doi:10.3390/healthcare12171732)
Supplement: Supplementary file 1 [file healthcare-12-01732-s001.zip › healthcare-3144681-supplementary.pdf]

SUPPLEMENTARY DATA

Table S1. The demographic data of participants by faculty and data collection year.

	All data collections n=996 (%)	Data collection 2015/16 (n=323)	Data collection 2017/18 (n=339)	Data collection 2019/20 (n=334)	p*
Faculty of Medicine					0.621
Ljubljana	652 (65.5)	206 (63.8)	221 (65.2)	225 (67.4)	
Maribor	344 (34.5)	117 (36.2)	118 (34.8)	109 (32.6)	

* chi-square test

Table S2. The demographic data of participants by faculty for the 2015/16 data collection year.

	Both faculties n=323 (%)	Faculty of Ljubljana n=206 (%)	Faculty of Maribor n=117 (%)	p
Gender				0.385#
Female	222 (68.7)	138 (67.0)	84 (71.8)	
Male	101 (31.3)	68 (33.0)	33 (28.2)	
Year of Study				0.200#
4th	183 (56.7)	111 (53.9)	72 (61.5)	
6th	140 (43.3)	95 (46.1)	45 (38.5)	
Environment (at time of high school)				0.227#
Urban	210 (65.0)	139 (67.5)	71 (60.7)	
Rural	113 (35.0)	67 (32.5)	46 (39.3)	
Siblings				0.170#
Yes	267 (82.7)	175 (85.0)	92 (78.6)	
No	56 (17.3)	31 (15.0)	25 (21.4)	
Father's Level of Education				0.375*
Elementary School	8 (2.5)	5 (2.4)	3 (2.6)	
High School	106 (32.8)	62 (30.1)	44 (37.6)	
University and higher	209 (64.7)	139 (67.5)	70 (59.8)	
Mother's Level of Education				0.119*
Elementary School	10 (3.1)	4 (1.9)	6 (5.1)	
High School	74 (22.9)	43 (20.9)	31 (26.5)	
University and higher	239 (74.0)	159 (77.2)	80 (68.4)	

* chi-square test, # Fisher's exact test

Table S3. The demographic data of participants by faculty for the 2017/18 data collection year.

	Both faculties n=339 (%)	Faculty of Ljubljana n=221 (%)	Faculty of Maribor n=118 (%)	p
Gender				0.214#
Female	238 (70.2)	150 (67.9)	88 (74.6)	
Male	101 (29.8)	71 (32.1)	30 (25.4)	
Year of Study				0.821#
4th	178 (52.5)	115 (52.0)	63 (53.4)	
6th	161 (47.8)	106 (48.0)	55 (46.6)	
Environment (at time of high school)				0.066#
Urban	232 (68.4)	159 (71.9)	73 (61.9)	
Rural	107 (31.6)	62 (28.1)	45 (38.1)	
Siblings				0.351#
Yes	285 (84.1)	189 (85.5)	96 (81.4)	
No	54 (15.9)	32 (14.5)	22 (18.6)	
Father's Level of Education				0.242*
Elementary School	13 (3.8)	7 (3.2)	6 (5.1)	
High School	111 (32.7)	67 (30.3)	44 (37.3)	
University and higher	215 (63.4)	147 (66.5)	68 (57.6)	
Mother's Level of Education				0.147*
Elementary School	9 (2.7)	5 (2.3)	4 (3.4)	
High School	92 (27.1)	53 (24.0)	39 (33.1)	
University and higher	238 (70.2)	163 (73.8)	75 (63.6)	

* chi-square test, # Fisher's exact test

Table S4. The demographic data of participants by faculty for the 2019/20 data collection year.

	Both faculties n=334 (%)	Faculty of Ljubljana n=225 (%)	Faculty of Maribor n=109 (%)	p
Gender				0.063#
Female	225 (67.4)	144 (64.0)	81 (74.3)	
Male	109 (32.6)	81 (36.0)	28 (25.7)	
Year of Study				0.907#
4th	180 (56.9)	122 (54.2)	58 (53.2)	
6th	154 (46.1)	103 (45.8)	51 (46.8)	
Environment (at time of high school)				0.140#
Urban	221 (66.2)	155 (68.9)	66 (60.6)	
Rural	113 (33.8)	70 (31.3)	43 (39.4)	
Siblings				1.000#
Yes	287 (85.9)	193 (85.8)	94 (86.2)	
No	47 (14.1)	32 (14.2)	15 (13.8)	
Father's Level of Education				0.096*
Elementary School	9 (2.7)	5 (2.2)	4 (3.7)	
High School	114 (34.1)	69 (30.7)	45 (41.3)	
University and higher	211 (63.2)	151 (67.1)	60 (55.0)	
Mother's Level of Education				0.376*
Elementary School	6 (1.8)	4 (1.8)	2 (1.8)	
High School	91 (27.2)	56 (24.9)	35 (32.1)	
University and higher	237 (71.0)	165 (73.3)	72 (66.1)	

* chi-square test, # Fisher's exact test

Table S5. Big Five Values: 2015/16 data collection raw values compared to the reference sample.

	Data collection 2015/16 (T values) n=323		Data collection 2015/16 (raw values) n=323		Reference sample 18-29 years old (17) (raw values) n=1445		p*
	M	SD	M	SD	M	SD	
Enthusiasm	46.0	9.7	41.0	6.2	43.2	6.2	<0.001
Assertiveness	45.5	8.8	36.8	6.0	39.8	6.7	<0.001
Compassion	48.1	9.5	44.6	4.9	45.2	5.1	0.054
Politness	46.6	9.9	40.8	5.7	42.2	5.6	<0.001
Orderliness	52.2	10.0	42.9	6.8	41.3	6.7	<0.001
Industriousness	47.9	8.6	45.8	5.5	46.8	6.3	0.008
Calmness	45.7	9.6	38.4	7.8	42.0	7.8	<0.001
Confidence	48.1	10.0	36.2	6.5	37.5	6.9	0.002
Openness	47.5	9.2	43.7	5.8	45.2	6.2	<0.001
Intellect	44.3	9.9	42.4	5.4	45.2	5.3	<0.001
**Extraversion**	45.4	9.2	77.8	10.6	83.0	11.5	<0.001
**Agreeableness**	47.4	9.9	85.4	9.4	87.4	9.4	<0.001
**Conscientiousness**	50.4	9.3	88.7	10.6	88.1	11.3	0.383
**Emotional stability**	46.7	9.3	74.6	12.8	79.6	13.5	<0.001
**Openness**	46.1	9.1	86.1	9.5	90.4	10.1	<0.001
L-score	49.2	8.2	33.3	6.1	33.8	7.4	0.175

* independent samples T-test

Table S6. Big Five Values: 2017/18 data collection raw values compared to the reference sample.

	Data collection 2017/18 (T values) n=339		Data collection 2017/18 (raw values) n=339		Reference sample 18-29 years old (17) (raw values) n=1445		p*
	M	SD	M	SD	M	SD	
Enthusiasm	45.3	9.9	40.5	6.5	43.2	6.2	<0.001
Assertiveness	44.6	9.3	36.2	6.4	39.8	6.7	<0.001
Compassion	48.5	9.4	44.9	4.9	45.2	5.1	0.326
Politness	46.4	9.8	40.7	5.5	42.2	5.6	<0.001
Orderliness	51.4	10.2	42.4	6.9	41.3	6.7	0.007
Industriousness	47.2	9.2	45.3	5.8	46.8	6.3	<0.001
Calmness	45.1	9.8	37.9	7.9	42.0	7.8	<0.001
Confidence	46.4	9.6	35.0	6.6	37.5	6.9	<0.001
Openness	47.9	9.2	43.9	5.8	45.2	6.2	<0.001
Intellect	42.8	9.7	41.6	5.4	45.2	5.3	<0.001
**Extraversion**	44.6	9.4	76.7	11.1	83.0	11.5	<0.001
**Agreeableness**	47.5	9.7	85.6	9.2	87.4	9.4	0.002
**Conscientiousness**	49.5	9.4	87.6	10.7	88.1	11.3	0.459
**Emotional stability**	45.5	9.4	72.9	13.2	79.6	13.5	<0.001
**Openness**	45.5	9.2	85.5	9.4	90.4	10.1	<0.001
L-score	47.3	8.0	31.9	5.9	33.8	7.4	<0.001

* independent samples T-test

Table S7. Big Five Values: 2019/20 data collection raw values compared to the reference sample

	Data collection 2019/20 (T values) n=334		Data collection 2019/20 (raw values) n=334		Reference sample 18-29 years old (17) (raw values) n=1445		p*
	M	SD	M	SD	M	SD	
Enthusiasm	44.3	10.1	39.9	6.5	43.2	6.2	<0.001
Assertiveness	43.8	8.8	35.6	6.0	39.8	6.7	<0.001
Compassion	47.5	10.2	44.3	5.5	45.2	5.1	0.004
Politness	46.1	10.8	40.4	6.3	42.2	5.6	<0.001
Orderliness	51.9	10.8	42.7	7.3	41.3	6.7	<0.001
Industriousness	46.8	8.9	45.1	5.6	46.8	6.3	<0.001
Calmness	44.2	9.8	37.1	8.1	42.0	7.8	<0.001
Confidence	46.3	10.6	34.9	7.2	37.5	6.9	<0.001
Openness	48.6	9.5	44.3	6.0	45.2	6.2	0.016
Intellect	44.4	10.1	42.5	5.4	45.2	5.3	<0.001
**Extraversion**	43.5	9.0	75.5	10.4	83.0	11.5	<0.001
**Agreeableness**	46.8	10.9	84.7	10.8	87.4	9.4	<0.001
**Conscientiousness**	49.7	9.9	87.8	11.2	88.1	11.3	0.661
**Emotional stability**	44.9	10.0	72.0	14.1	79.6	13.5	<0.001
**Openness**	46.8	9.3	86.9	9.4	90.4	10.1	<0.001
L-score	46.6	8.2	31.3	6.2	33.8	7.4	<0.001

* independent samples T-test

REFERENCES

35. Caprara, G.V.; Barbaranelli, C.; Borgogni, L.; Bucik, V.; Boben, D.; Hruševar-Bobek, B.; Zupančič, M.; Horvat, M. Model “Velikih Pet”: Pripomočki za Merjenje Strukture Osebnosti: Priročnik; Center za psihodiagnostična sredstva: Ljubljana, Slovenia, 2012.
